# Are Current Patient-Reported Outcome Measures Fit for Purpose to Evaluate Unicompartmental Knee Arthroplasty?

**DOI:** 10.3390/jcm14010203

**Published:** 2025-01-02

**Authors:** John M. Bayram, Nicholas D. Clement, Andrew J. Hall, Phil Walmsley, Jon V. Clarke

**Affiliations:** 1 Department of Orthopaedics, Golden Jubilee University National Hospital, Glasgow G81 4DY, UK; 2 Edinburgh Orthopaedics, Royal Infirmary of Edinburgh, Edinburgh EH16 4SA, UK; 3 School of Medicine, University of St Andrews, St Andrews KY16 9TF, UK

**Keywords:** medial compartment osteoarthritis, unicompartmental knee arthroplasty (UKA), total knee arthroplasty (TKA), robotic-assisted arthroplasty, patient-reported outcome measures (PROMs), outcome assessment, ceiling effects

## Abstract

The optimal procedure for isolated end-stage medial compartment knee osteoarthritis (OA) remains uncertain, with debate persisting between unicompartmental knee arthroplasty (UKA) and total knee arthroplasty (TKA). The aim of this narrative review is to evaluate current outcome measures in knee arthroplasty (KA) and explore how evolving patient populations and technological advancements may necessitate the use of different patient-reported outcome measures (PROMs) for evaluating UKA. While UKA offers potential advantages over TKA in early pain relief and functional outcomes, most randomised control trials using traditional PROMs have failed to show definitive superiority. The recent introduction of robotic assistance may have further enhanced the benefits of UKA. However, it remains uncertain whether the advantages outweigh the higher revision rates associated with UKA. Although traditional PROMs, such as the Oxford Knee Score or Knee Injury and Osteoarthritis Outcome Score, were designed for the KA population of 30 years ago, they continue to be employed today. The current KA population, particularly those undergoing UKA, are typically younger, physically fitter, and have higher functional demands than those for whom traditional PROMs were originally designed. As a result, these PROMs are now limited by ceiling effects. High-performance PROMs, such as the Forgotten Joint Score-12 or the metabolic equivalent of task score, have recently been utilised for high-demand patients and do not have postoperative ceiling effects. Return to work and sport are also important outcomes that are often overlooked for younger, high-demand patients. Future studies should aim to define the differences between UKA and TKA populations, identify patient factors that predict UKA success, and validate high-performance PROMs for UKA. This will provide deeper insights into the functional benefits of UKA and TKA, enabling patients and surgeons to make more informed decisions regarding implant selection.

## 1. Introduction

There is an ongoing debate as to whether unicompartmental knee arthroplasty (UKA) is superior to total knee arthroplasty (TKA) for the treatment of isolated end-stage medial compartment knee osteoarthritis (OA) [1]. Over the past decade, UKA has experienced a resurgence in countries such as the United Kingdom (UK), Canada, Australia, Sweden, and Norway, with usage rates currently ranging from 7.3% to 14.5% of all primary knee arthroplasties (KA) [2,3,4,5,6]. In contrast, the use of UKA declined in the United States of America (USA) during this period [7]. More recently, however, evidence indicates a growing adoption of UKA within American healthcare systems [8]. Up to 47.9% of patients with knee OA have been reported to be radiologically suitable for UKA [9]. However, when applying the 2016 American Academy of Orthopaedic Surgeons (AAOS) appropriate use criteria [10], which consider clinical context and are based on expert consensus, this proportion drops to 13.1% [11]. The National Institute for Health and Care Excellence (NICE) currently recommends offering patients with isolated medial compartment OA in the UK a choice between UKA and TKA [12]. However, there remains no clear consensus on which patient groups are best suited for each procedure.

The 2022 AAOS guidelines summarise the evidence comparing UKA with TKA for patients with medial compartment knee OA, reporting that UKA offers similar patient-reported outcome measure (PROM) scores for pain and function, with some evidence suggesting superior outcomes in the short to mid-term [13]. UKA is also associated with a shorter operative time, lower blood loss, lower transfusion rates, shorter length of hospital stay, lower complication rates, greater range of motion, and higher levels of activity. UKA patients return to work sooner and at a higher rate than those undergoing TKA [14,15,16], and they also achieve higher rates of return to sports [17]. UKA is also potentially more cost-effective up to 5 years postoperatively compared to TKA [18]. The procedure is less invasive and preserves bone stock, which can simplify future revisions. Additionally, by preserving the anterior cruciate ligament (ACL), lateral meniscus, and surrounding soft-tissue envelope, it more effectively retains natural knee kinematics [19,20]. The main concern for UKA is higher overall revision rates, especially at long-term follow-up [2,3,4,5,6,7]. The AAOS guidelines concluded that further research should aim to identify the optimal patient subset for which UKA would be recommended for greatest survivorship and functional benefit. The purpose of this narrative review was to assess current outcome measures in KA and to explore why different PROMs may be necessary when evaluating UKA. The review is organised into a ‘conceptual frame’ with four key sections [21]: core outcomes domains, changes in the UKA population, PROMs employed for UKA, and the technological evolution of UKA.

## 2. Core Outcome Domains for Knee Arthroplasty

### 2.1. Core Outcome Domains

The James Lind Alliance Priority Setting Partnership’s current number one priority in KA research is determining the most important patient and clinical outcomes, and the best way to measure them [22]. To comprehensively assess the differences between UKA and TKA outcomes, it is critical to ensure the correct outcome measures are being employed. The International Consortium for Health Outcomes Measurement (ICHOM) defined four core outcome domains for assessing outcomes in knee OA: joint pain, joint function, health-related quality of life (HRQoL), and work status [23]. The Outcome Measures in Rheumatology (OMERACT) and Osteoarthritis Research Society International (OARSI) also defined a core domain set that are mandatory in all knee OA clinical trials: pain, physical function, quality of life, patient’s global assessment of the joint, and adverse events (Table 1) [24]. Apart from adverse events, these are all typically assessed using PROMs. Joint and physical function can also be assessed using three-dimensional motion capture or performance-based measures, such as the Timed Up and Go test [25]. Although these techniques can provide valuable insights into objective functional changes not detected by PROMs [26], there is no consensus on the best objective physical measure of “function” after KA [27]. Furthermore, most physical tests are resource-intensive and fail to capture the patient’s subjective experience, making them unsuitable as replacements for PROMs in routine outcome assessment.

### 2.2. Health-Related Quality of Life

HRQoL is typically measured with PROMs that are validated across multiple health conditions and are therefore suitable for health economic (cost-utility) analysis, such as the EuroQoL-5 Dimension (EQ-5D) [28]. Although the EQ-5D is not joint-specific, it does show a large effect size following KA [29]. The 3-level EQ-5D (EQ-5D-3L) is however limited by a postoperative ceiling effect of 29.8%. Despite its limitations when measuring condition-specific HRQoL [30] the EQ-5D remains the gold standard HRQoL measure [31]. Knee-specific HRQoL measures also exist, such as the Knee Quality of Life-26 (KQOL-26) [32], OsteoArthritis Knee and Hip Quality Of Life (OAKHQOL) [33], and OsteoArthritis-Quality of Life (OA-QoL) [34]. These are more responsive than generic HRQoL measures, but their lack of ability to compare health economics between different health conditions limits their utility. ICHOM currently recommends the use of the EQ-5D-3L, the 12-Item Short Form Health Survey (SF-12) or the Veterans RAND 12 Item Health Survey (VR-12) for HRQoL assessment [23]. NICE recommends the EQ-5D-3L for cost-utility analyses, but also states that other measures may be needed if key dimensions of health are not captured for a given patient population.

### 2.3. Joint-Specific Function and Pain

Joint-specific function and pain are typically measured with knee-specific PROMs. Reynaud et al. [35] conducted a systematic review assessing the psychometric qualities of PROMs for TKA clinical trials. The Oxford Knee Score (OKS), Knee Injury and Osteoarthritis Outcome Score (KOOS) and Western Ontario and McMaster Universities Osteoarthritis Index (WOMAC) were the only joint-specific PROMs deemed to have undergone an extensive validation process for TKA. Despite this, even the OKS, KOOS and WOMAC were of ‘doubtful’ quality in some psychometric qualities, such as test–retest agreement. More recently introduced high-performance PROMs, such as the Forgotten Joint Score-12 (FJS-12) were not evaluated in this review. It also only assessed PROMs with classical test theory, without incorporating more advanced methods such as item response theory (IRT). The OKS and FJS-12 have, however, recently been validated using IRT by Khatri et al. [36,37]. Wang et al. [38] also reviewed the psychometric properties of knee-specific PROMs. They identified nine PROMs that met the minimum criteria for psychometric validation, but reported that none of them had been validated for all measurement properties. It is likely that a PROM needs to be well established over many years before the necessary psychometric assessments can be performed, and that more modern scores have not been in use for long enough to be fully validated. The OKS and KOOS are the knee-specific PROMs predominantly used by international arthroplasty registries to audit performance [2,3,4,5,6,7]. ICHOM currently recommends the use of the KOOS-Physical Function Shortform (KOOS-PS) for measuring joint function and a visual analogue scale (VAS) or numeric rating scale (NRS) for joint pain [22].

### 2.4. Revision as an Outcome Measure

Revision is a critical outcome measure in KA, and its consideration is essential when evaluating the suitability of UKA compared to TKA. The International Society of Arthroplasty Registries uses revision as an outcome because it is ‘an unambiguous endpoint that indicates that both the surgeon and the patient are unhappy with the outcome of the primary procedure’ [39]. Although the ideal implant would aim to eliminate the need for revision, the limited longevity of current implants means that revision is likely if a patient lives long enough. The National Joint Registry (NJR) of England, Wales, Northern Ireland and the Isle of Man 2023 report shows that, at various timepoints between 1 and 19 years after primary UKA, the revision risk is more than double that of TKA [2]. In contrast, some high-volume centres have reported UKA survivorship rates comparable to those of TKA [40], and surgeons with the highest UKA usage (>30% of their practice) have much lower revision rates than low-usage surgeons [41].

There are many factors that contribute to the high revision rates for UKA. Firstly, there appears to be a lower revision threshold for UKA compared to TKA [42,43]. For example, one study found that among patients with an OKS below 20 (indicating a very poor outcome), only 12% of TKAs were revised, compared to 63% of UKAs [42]. This may be because UKA is a less invasive procedure that preserves more bone stock, potentially making it technically easier to revise than TKA. Additionally, unexplained pain is almost seven times more likely to be managed with revision in UKA compared to TKA [44]. Unexplained pain therefore contributes significantly to the revision burden of UKA, but does not for TKA, despite being equally as prevalent [45]. Both UKAs and TKAs revised for unexplained pain have poorer outcomes than those revised for other indications [46,47,48]. The most common mode of failure in UKA is progressive arthritis, which is an additional mode of failure when comparing to TKA. It is also important to recognise that revisions can vary in complexity. Revision as a result of prosthetic joint infection (PJI) often requires staged procedures, prolonged hospital stays, and results in poor functional outcomes [49]. This is unsurprisingly associated with substantial financial costs [50]. TKA has a significantly higher PJI-related revision risk compared to UKA, with some UKA studies reporting zero revisions attributable to PJI [51,52]. Outcomes following revision also vary by context. While some studies report that patients undergoing UKA to TKA revision achieve PROM scores comparable to primary TKA [53,54], others suggest their outcomes are more aligned with those of revision TKA [55,56].

The NJR 2023 report shows that younger age groups are more likely to require revision at all timepoints than older age groups, and that UKA patients are a median of 5 years younger than TKA patients [2]. Female sex and higher American Society of Anesthesiologists (ASA) grades are also known risk factors for UKA revision [57]. For younger KA patients, lifetime revision risk will always be higher due to their remaining lifespan at the time of surgery [58]. Revision is, therefore, an anticipated outcome and might only be considered a ’failure’ if it occurs earlier than expected. Some patients may find the higher revision risk of UKA to be an acceptable trade-off for its various potential benefits. Compared to TKA, UKA may enable patients to remain more active, maintain better fitness, continue working, and enjoy a more fulfilling life for longer before potentially requiring conversion to a TKA. Revision rates should therefore not be interpreted in isolation, as indication and context of revision are critical when comparing UKA and TKA.

## 3. Changes in the UKA Population

### 3.1. Knee Arthroplasty Population Changes

The OKS, KOOS, and WOMAC have all been shown to exhibit postoperative ceiling effects depending on the methods of assessment used [59,60]. These scoring systems were all originally developed in the late 20th century: the OKS and KOOS in 1998 [61,62], and the WOMAC in 1982 [63]. They were all designed for the knee OA population of the 1980s and 1990s (Table 2). The knee OA population of today is higher functioning than that of three decades ago. Between 1998 and 2024, mean pre-operative OKS scores for TKA patients have increased from 16.4 to 20.2, while post-operative scores have risen from 30.7 to 36.6 [61,64]. UKA patients today have been shown to have even higher pre- (21.3) and post-operative OKS scores (44.5) [65]. These differences in scores exemplify the importance of revalidating these traditional PROMs for today’s TKA population. Similarly, UKA patients represent a distinct group, necessitating separate validation. This underscores the need to ensure that PROMs are validated specifically for the populations in which they are being applied, particularly when they differ from the original validation cohort.

The proportion of patients undergoing KA at a younger age is rising, with approximately 15% of all primary KA patients now being under 60 years old [66]. Between 2004 and 2014, the number of KA patients aged 45 to 64 in the USA increased by 188%, compared to an 89% increase in those aged 65 to 84 [67]. This younger population has higher levels of physical function and expects to return to similar levels post-operatively [68]. Higher-demand patients are more likely to achieve a ceiling score for these traditional PROMs, which primarily focus on pain and activities of daily living (ADLs) [69]. This group of patients at the top of the scoring scale remain undifferentiated from those who function at a higher level (e.g., partake in sport) and those who are limited to basic ADLs. One example of this is a study comparing different TKA implants that showed no difference in OKS, WOMAC or Knee Society Score (KSS) [70], but did show significant improvements in a high-performance PROM, the Total Knee Function Questionnaire (TKFQ) [71], which assesses activities like kneeling, gardening, swimming, and golf [72].

### 3.2. UKA Indication Changes

In 1989, Kozinn and Scott’s landmark paper described UKA as being most appropriate for low-demand patients over the age of 60 [73]. Indications have since broadened significantly, and UKA is now considered safe, effective, and perhaps optimal for those under 60 or with high functional demands [74]. There is, however, still no agreement whether age and functional status should be used for UKA patient selection. Exactly how patients who are eligible for UKA (medial compartment OA with an intact ACL) differ from those for whom TKA is the only surgical option (e.g., tricompartmental OA) also remains unclear. Isolated medial compartment OA can be a precursor to multi-compartmental OA, so it is unsurprising that many patients who are technically appropriate for UKA are younger than patients with multi-compartmental OA who require TKA [2]. UKA patients have a lower body mass index (BMI) and are less comorbid than TKA patients (Table 3) [2], suggesting they may be physically fitter. Conversely, some are advocating for the use of UKA in comorbid patients, when technically appropriate, to reduce their operative risks [75]. This diversity in the UKA population highlights the challenge in interpreting clinical trial results without knowledge of the patient selection criteria employed.

### 3.3. Patient Predictors of Success in UKA

The evidence on which patient groups achieve the best outcomes in UKA is limited. Thompson et al. [76] found that patients under 60 years old had better KSS scores at two years post-operatively than those aged 60 or older. Zuiderbaan et al. [77] also found that patients under 65 years old experienced greater improvements in total WOMAC and in the pain sub-scale compared to those aged 65 or older. Other studies have not found age to predict PROM scores [78,79]. Sex, BMI, ASA grade, and comorbidity have also not been found to be associated with PROM scores [77,78,79]. However, these findings are potentially influenced by selection bias, as certain patients may have been considered unsuitable for UKA based on these factors. Both pre-operative and post-operative lower limb alignment measurements, such as mechanical axis and hip–knee–ankle angle, have also been shown to predict PROM scores [77,79]. As with TKA [80], poor mental health scores are predictive of low satisfaction in UKA [81]. It seems likely that patients who achieve high PROM scores following UKA, a younger and likely higher-demand group, would also achieve good scores if they were to have a TKA instead. Indeed, having a high-scoring pre-operative PROM “phenotype” is associated with satisfaction after TKA [82]. Defining this high-demand group remains a challenge; however, clinic-based fitness tests for patients with arthritis may offer a novel and practical method for identification [83]. Although high-demand patients appear to achieve good outcomes with UKA, it remains uncertain whether UKA is superior to TKA for this subgroup.

## 4. Patient-Reported Outcomes in UKA

### 4.1. Randomised Control Trials

Randomised control trials (RCTs) are the gold standard for comparing interventions and, when incorporating PROMs, provide critical evidence to guide implant selection in KA. Current RCT evidence suggests non-significant differences in PROM scores between UKA and TKA. The minimal clinically important difference (MCID) has been used to determine if differences in PROM scores are meaningful to patients, but improving current KA outcomes above the MCID would mean an increase in most scores of over 25% [84]. It has been suggested that this could be near-impossible to achieve, and that repeated measures assessment during follow-up could reduce the MCID to a more achievable 5% to 10%. Bin et al. [85] conducted a systematic review and meta-analysis of surgical interventions for knee OA, identifying eight RCTs that compared PROM scores between UKA and TKA. The most used PROMs were OKS (*n* = 5) and KSS (*n* = 4). The KOOS, WOMAC, and FJS-12 were each used once. Only one of the eight RCTs definitively showed superiority in PROMs for UKA [86]. This was the only study to use a high-performance PROM without an observed ceiling effect, the FJS-12.

### 4.2. High-Performance PROMs

High-performance PROMs, such as the FJS-12, TKFQ, the High Activity Arthroplasty Score (HAAS) [69], and the metabolic equivalent of task (MET) score [87], have been designed to evaluate outcomes in high-demand KA patients. The University of California, Los Angeles (UCLA) Activity Score is another PROM first described in 1984 that assesses higher functioning [88]. These high-performance PROMs have no observed ceiling effects and are better at discriminating between high-demand patients. Notably, some traditional PROMs have been updated to assess higher functional demands as well. The KSS was revised in 2012 [89], and the OKS can now be supplemented with the OKS-Activity and Participation Questionnaire (OKS-APQ) [90]. Most high-performance PROMs are, however, yet to be used in a UKA versus TKA RCT. Harrison et al. [91] used IRT to map the OKS and HAAS onto a common scale, which allows for pooling of results between studies that use either PROM. This approach could allow for comparison between previous RCTs and new trials that use high-performance PROMs. High-performance PROMs could more accurately differentiate outcomes between UKA and TKA, particularly in high-demand patients.

### 4.3. Return to Work

Returning to work is important to working-age KA patients [92]. This population is expanding both due to increasing retirement ages and increasing KA usage in patients under 65 years old [67]. Many people need to work to maintain their social and financial wellbeing. Work is also important for the economy; the cost of working days lost due to arthritis in the UK was estimated at GBP 2.58 billion in 2017, and is expected to rise to GBP 3.43 billion by 2030 [93]. Approximately 82% of KA patients return to work at an average of three months post-operatively [94]. UKA patients have been reported to return to work sooner than TKA patients, with average return to work in as little as 3 to 6 weeks [14,15,16]. Only 3% of UKA patients report that their ability to return to work is significantly worse post-operatively, compared to 12% for TKA [95]. UKA patients also report lower rates of knee-related job loss (9%) compared to TKA patients (17%) [16]. Despite it being one of ICHOM’s four core outcome domains for knee OA [23], return to work is often overlooked as an outcome in clinical trials.

### 4.4. Return to Sport

Return to sport after KA is also an increasing priority for many patients. The current consensus is that patients can return to low-impact sports (e.g., cycling, golf) following UKA or TKA [96]. Moderate-impact sports (e.g., hiking, downhill skiing) can be returned to with prior experience, and high-impact sports may not be recommended. Both young and elderly UKA patients have been shown to achieve a higher and faster return to sport rate than TKA patients [97,98]. TKA has a median return to sport rate of 71.2%, whereas UKA consistently achieves a rate of 80% to 93% [99,100]. Additionally, 98% of patients who participate in vigorous or moderate-intensity sports pre-operatively have been shown to return to this level of activity following UKA [101]. UKA may therefore be preferred to TKA in high-demand patients for whom returning to work or sports is a priority.

## 5. The Evolution of UKA

### 5.1. Advancements in UKA Technology

While UKA has undergone significant advancements over the past two decades and continues to evolve, traditional PROMs have not kept pace with these innovations. There are currently at least 16 different UKA implants on the market, with various tibial bearing designs, fixation techniques, alignment strategies, and robotic or navigation assistive technologies [102]. As already described, UKA patient selection is also continuing to evolve, with increased attention on the excellent outcomes in younger patients over recent years [74]. With these ongoing changes in UKA, many of the UKA versus TKA RCTs assessed by Bin et al. [85] are unlikely to reflect current practice. Additionally, surgeon volume and usage of manually performed UKA (mUKA), a risk factor for malpositioning and early failure [41,52], are seldom reported in clinical trials. RCTs using robotic-assisted UKA (RAUKA), which has improved precision in component placement compared to mUKA [103], are indicated to mitigate these factors.

### 5.2. Robotic-Assisted UKA

RAUKA has been rapidly adopted since Mako Surgical implanted the first prosthesis in 2006 [104]. The improved precision in component positioning seen in RAUKA may be achieved from the outset without the associated learning curves of traditional manual techniques [103]. This may result in a lower malpositioning and early failure rates in RAUKA than those observed in mUKA. Malpositioning in mUKA occurs more frequently during the learning curve of the procedure and is the main cause of failure overall [41,52]. Blyth et al. [105] found that RAUKA was associated with better early pain relief and improved functional outcomes compared to mUKA. A higher pre-operative UCLA Activity Score and the use of RAUKA were linked to excellent KSS scores, while mUKA and depression were correlated with worse scores. Additionally, a greater proportion of patients in the RAUKA group showed improvement in their UCLA Activity Score compared to those in the mUKA group. Patients may also be able to return to work sooner and to sport at higher rates following RAUKA compared to mUKA [99,106]. Improved PROM scores following RAUKA has been attributed to the robot’s ability to achieve precise reconstruction of joint surfaces, enhanced alignment, and more natural knee kinematics [105]. It appears that RAUKA could be cost-effective or cost-neutral when compared to mUKA, despite the costs associated with pre-operative computed tomography (CT) scans and the robot itself, primarily due to reduced hospital length of stay [107]. High-performance PROMs could be necessary to differentiate outcomes between RAUKA and mUKA, as traditional PROMs may lack the sensitivity to capture the outcome improvements associated with advancements in UKA technology.

## 6. Conclusions

UKA has demonstrated potential advantages over TKA in terms of early pain relief, faster recovery, and improved functional outcomes. However, these benefits are unlikely to be fully captured when employing traditional PROMs. The contemporary KA population is typically younger, more physically fit, and has higher functional demands compared to the population for which these traditional PROMs were originally designed. Further investigation is required to better understand the specific differences between KA patients who are eligible for UKA and those who are not. There may be an optimal subset of UKA-eligible patients, potentially defined by higher levels of function, that would benefit the most from UKA. Future studies investigating predictors of success in UKA, possibly incorporating clinic-based fitness tests for patients with arthritis [83], may help identify this group. High-performance PROMs, offering greater sensitivity and no ceiling effects, are needed to accurately evaluate the differences in outcome between UKA and TKA. This could also be true for the assessment of other technological advancements in KA, such as robotic-assisted surgery. Return to work and sport are other outcomes that are important to high-demand patients but remain underutilised in clinical trials. The development, validation and utilisation of high-performance PROMs will enhance understanding of functional benefits following UKA and TKA. This will enable patients and surgeons to make more informed decisions about implant choice in the context of different revision risk trajectories.

## Figures and Tables

**Table 1:** The International Consortium for Health Outcomes Measurement (ICHOM) core outcome domains and the Outcome Measures in Rheumatology (OMERACT) and Osteoarthritis Research Society International (OARSI) core domain set for knee osteoarthritis (OA).

ICHOM Core Outcome Domains for Knee OA	OMERACT-OARSI Core Domain Set for Knee OA Clinical Trials
Joint pain	Pain
Joint function	Physical function
Health-related quality of life	Quality of life
Work status	Patient’s global assessment of the joint
	Adverse events

**Table 2:** The original design intentions for traditional knee-specific patient-reported outcome measures in the authors’ words.

Patient-Reported Outcome Measure (Year of Publication)	Original Design Intention in the Authors’ Words
Oxford Knee Score (OKS; 1998)	“The development of a short questionnaire for use in total knee replacement (TKR) which was designed to be reliable, reproducible, valid and sensitive to change, with a high rate of completion.” [57]
Knee Injury and Osteoarthritis Outcome Score (KOOS; 1998)	“At the time, no patient reported outcome measures (PROMs) existed for younger or more physically active individuals with knee osteoarthritis (OA), and no existing PROMs could be used from the time of knee injury to severe OA. We, therefore, set out to develop a PROM for this purpose.” [58]
Western Ontario and McMaster Universities Osteoarthritis Index (WOMAC; 1982)	“To build a standardized disease-specific patient-relevant self-reported health status questionnaire for hip and knee osteoarthritis (OA)” [59]

**Table 3:** Total number of total knee arthroplasty (TKA) and unicompartmental knee arthroplasty (UKA) procedures recorded in the The National Joint Registry (NJR) of England, Wales, Northern Ireland and the Isle of Man from 2018 to 2022 with American Society of Anesthesiologists (ASA) grades and age groups (in years) [2].

	Procedures		ASA Grade					Age Group				
	*n*	%	ASA1	ASA2	ASA3	ASA4/5	ASA1:ASA3	<60	60–69	70–79	>80	<60:>70
2022	101,995											
TKA	88,163	86	5%	73%	22%	<1%	0.24	13%	31%	40%	14%	0.25
UKA	13,832	14	13%	76%	12%	<1%	1.08	27%	34%	28%	7%	0.77
2021	83,144											
TKA	71,648	86	6%	72%	21%	<1%	0.31	14%	30%	39%	14%	0.27
UKA	11,496	14	14%	75%	11%	<1%	1.24	29%	34%	28%	6%	0.84
2020	54,188											
TKA	46,915	87	7%	73%	20%	<1%	0.37	15%	31%	39%	13%	0.28
UKA	7273	13	16%	74%	10%	<1%	1.68	30%	33%	27%	6%	0.91
2019	110,814											
TKA	97,955	88%	7%	72%	21%	<1%	0.31	14%	31%	39%	14%	0.27
UKA	12,859	12%	16%	73%	11%	<1%	1.43	31%	33%	27%	7%	0.92
2018	106,845											
TKA	94,866	89%	7%	72%	20%	<1%	0.36	14%	31%	39%	14%	0.27
UKA	11,979	11%	17%	73%	10%	<1%	1.63	32%	33%	26%	7%	0.97

## Data Availability

Not applicable.
